# Comparative Effects of Ce, Co, or W Dopants on the Catalytic Performance of FeMnTiO_x_ Catalysts for Low-Temperature NH_3_-SCR of NO

**DOI:** 10.3390/molecules31162775

**Published:** 2026-08-10

**Authors:** Binyu Wang, Cong Feng, Huan Liu

**Affiliations:** 1 State Key Laboratory of Clean and Efficient Coal Utilization, Taiyuan University of Technology, Taiyuan 030024, China; 2 Key Laboratory of Energy Conversion Photoelectronic Functional Materials, School of Mathematics and Physics, Jiangxi Education Institutes, Jinggangshan University, Ji’an 343009, China

**Keywords:** NH_3_-SCR, FeMnTiO_x_ catalyst, Ce doping, low-temperature denitration, redox property

## Abstract

To clarify the role of metal dopants in low-temperature NH_3_-SCR of NO, FeMnTiO_x_ catalysts were synthesized by coprecipitation and separately modified with Ce, Co, or W. The catalysts were evaluated for NO conversion, separate O_2_/H_2_O switching response and dry-feed time-on-stream stability and characterized by XRD, SEM, N_2_ adsorption–desorption, XPS, H_2_-TPR and NH_3_-TPD. Ce doping at Ce/Mn = 0.3 suppressed TiO_2_ crystallization, increased the BET surface area from 136 to 231 m^2^ g^−1^ and the pore volume from 0.19 to 0.58 cm^3^ g^−1^, raised the Mn^4+^/Mn^3+^ ratio from 0.70 to 1.25, and increased the medium-strong-acid relative peak area from 555.4 to 1293.2 a.u. FeMnCe_0.3_TiO_x_ maintained at least 90% NO conversion from 140 to 360 °C, gave 75% conversion at 400 °C, and averaged 97.4% during a 40 h dry-feed test at 350 °C. The results show that the superior Ce-modified catalyst arises from the combined regulation of texture, surface redox balance and acidity.

## 1. Introduction

With the continuous development of the social economy and the steady improvement of residents’ quality of life, all sectors of society are paying increasing attention to the quality of the living environment. Green development, environmental protection and sustainability have become important hallmarks of a sound ecological environment. At present, one of the main sources of environmental pollution is the combustion and utilization of fossil energy such as coal, petroleum and natural gas, as well as the thermal power generation process that uses various fuels as the primary energy source [1].

NO_x_ is a harmful byproduct of high-temperature combustion, formed by the combination of nitrogen and oxygen in the air. Legislation in most developed countries requires the removal of these oxides from exhaust gases [2]. At present, selective catalytic reduction (SCR) technology features high denitrification efficiency and good utilization, and has been industrialized worldwide. The most important component in SCR technology is the selection of a catalyst [3]. Precious metal catalysts are one category, including Pt [4], Pd [5], Rh [6], Au [7], generally supported on Al_2_O_3_, TiO_2_ and SiO_2_ [8]. Although precious metal catalysts show high efficiency in the reduction of NO_x_, they easily suffer from poisoning and deactivation, and their high cost is unfavorable for industrial application [9]. In view of this, non-precious metal catalysts have attracted extensive attention. Currently, the most commonly used catalyst system consists of V_2_O_5_ as the active component, titanium dioxide as the support, and molybdenum and tungsten as promoters. However, when V_2_O_5_/TiO_2_ is used as an SCR catalyst for flue gas denitrification, the operating temperature must be higher than 350 °C. Long-term exposure to high temperatures causes sintering of the active sites on the surface of the catalyst, leading to increased catalyst particle size, reduced specific surface area, and thus decreased catalytic activity [10].

Beyond catalyst composition, electrification of catalytic reactors has emerged as a complementary direction for deNO_x_ control. Induction heating catalysis (IHC) couples an alternating electromagnetic field with a susceptible metallic or ferromagnetic catalyst/support, thereby generating heat within the catalytic body and enabling fast start-up, localized energy delivery and reduced thermal inertia [11]. In a recent deNO_x_ demonstration, Pd/Re-modified NiMo structures designed for IHC achieved approximately 95% NO_x_ conversion at 250 °C and were proposed for cold-start exhaust treatment [12]. The FeMnMTiO_x_ powders in the present study were assessed in a conventionally heated fixed bed and are not presented as IHC-ready materials; nevertheless, IHC provides an important benchmark showing that intrinsic surface chemistry and electrically responsive heat management can be developed as complementary strategies.

Given the shortcomings of the aforementioned catalysts, MnO_x_ has been widely used in recent years owing to its excellent low-temperature activity. Moreover, manganese oxides with different valence states (Mn^4+^, Mn^3+^, Mn^2+^) possess strong electron transfer ability [13]. Li et al. [14] prepared a MnTi catalyst by ultrasonic impregnation for the simultaneous removal of NO. Performance evaluation results showed that the removal efficiencies reached 96% at a low temperature of 200 °C. Peña et al. [15]. investigated the low-temperature catalytic activity of first-row transition metal oxides supported on anatase TiO_2_. The results demonstrated that manganese-based catalysts presented the highest catalytic activity owing to their Lewis acidity, redox properties, and high surface concentration. However, the SCR reaction of NO over MnO_x_/TiO_2_ catalysts tends to generate N_2_O as a byproduct. In addition, the presence of SO_2_ in the flue gas still leads to deactivation of MnO_x_/TiO_2_ catalysts [16]. Therefore, the modification of manganese-based catalysts has become a research hotspot.

Studies have shown that the introduction of other elements can improve the catalytic performance to a certain extent. Song et al. [17] prepared Fe-, Cu-, Ce-, and Mn-supported NH_3_-SCR denitrification catalysts using natural red volcanic rock as the support, and determined AVR-10Mn-450 as the optimal catalyst. This catalyst exhibited an NO conversion exceeding 90% at 175 °C with a space velocity of 25,000 h^−1^, showing outstanding low-temperature activity. Its porous structure, Mn-Fe synergistic effect, as well as the abundant highly active adsorbed oxygen, high-valence metal species and medium-strong acid sites on the surface are the key reasons for its high activity. In addition, the catalyst shows good stability and resistance to SO_2_/H_2_O poisoning. The denitrification reaction follows a dominant E-R pathway assisted by the L-H pathway, and the Mn-Fe redox cycle further boosts the catalytic performance.

Although Ce, Co, and W are established modifiers for NH_3_-SCR catalysts, no claim of elemental novelty is made here. The novelty of this work lies in a controlled, head-to-head comparison in which each dopant is introduced into the same FeMnTiO_x_ matrix using an otherwise identical coprecipitation procedure. The Ce series provides a two-level loading comparison (Ce/Mn = 0.1 and 0.3), whereas Co and W at M/Mn = 0.3 isolate dopant-identity effects at the same upper loading. This design reduces the confounding effects of support, preparation route and test protocol that often complicate cross-study comparisons. By combining catalytic tests with XRD, SEM, N_2_ adsorption–desorption, XPS, H_2_-TPR and NH_3_-TPD, the study identifies how dopant-dependent changes in crystallinity, texture, redox balance and acidity jointly control activity and stability, rather than attributing performance to a single descriptor.

## 2. Results and Discussion

### 2.1. XRD Analysis

**Figure 1:**
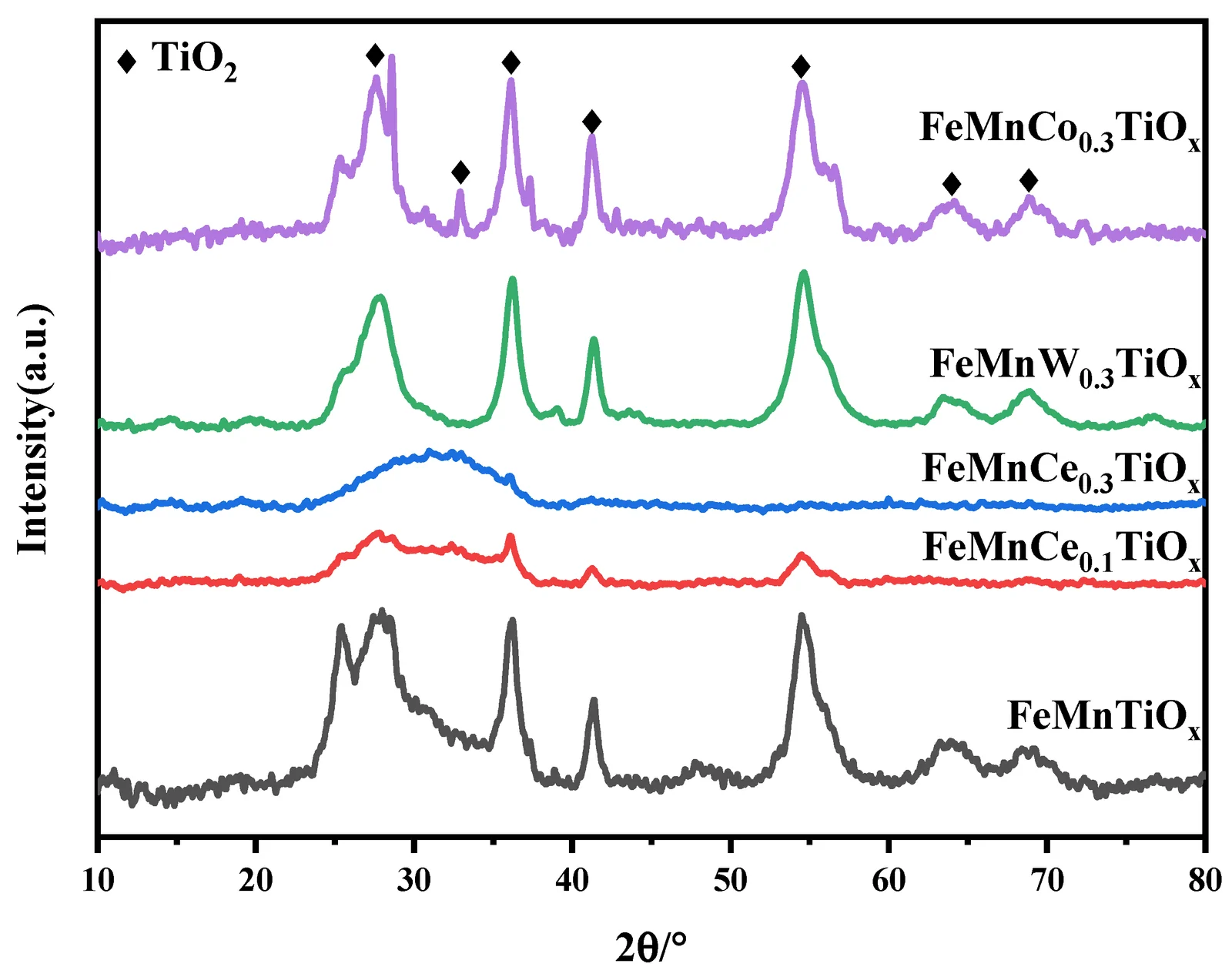
XRD patterns of FeMnTiO_x_ and FeMnMTiO_x_ catalysts (M = Ce, Co, W).

Figure 1 displays the XRD patterns of FeMnTiO_x_, FeMnCeTiO_x_ catalysts with different Ce/Mn molar ratios (0.1 and 0.3), and FeMnM_0.3_TiO_x_ catalysts (M = W or Co). Diffraction peaks at 27.4°, 36.1°, 41.2°, 44.0°, 54.3°, 56.6° and 69.8° are assigned to rutile TiO_2_, corresponding to the (110), (101), (111), (210), (211), (220) and (301) crystal planes, respectively [18,19]. No distinct FeO_x_, MnO_x_, CeO_x_, CoO_x_ or WO_x_ reflections are resolved. This observation is consistent with, but does not by itself prove, high dispersion or poor crystallinity; nanocrystalline domains and phases below the XRD detection limit cannot be excluded [20]. At Ce/Mn = 0.1, the TiO_2_ reflections weaken, whereas at Ce/Mn = 0.3 they are largely replaced by a broad halo, suggesting that Ce incorporation suppresses TiO_2_ crystallite growth and promotes a more disordered oxide matrix [21]. This qualitative interpretation is consistent with the larger surface area and pore volume measured by N_2_ adsorption–desorption [22,23]. Co- and W-containing samples do not show a resolvable peak shift, and their sharper TiO_2_ features suggest greater crystallinity than FeMnCe_0.3_TiO_x_. Because the patterns contain broad and overlapping features, no Rietveld refinement or quantitative phase fraction is reported, and the XRD discussion is restricted to qualitative phase assignment and relative crystallinity.

### 2.2. SEM and EDS Mapping Analysis

**Figure 2:**
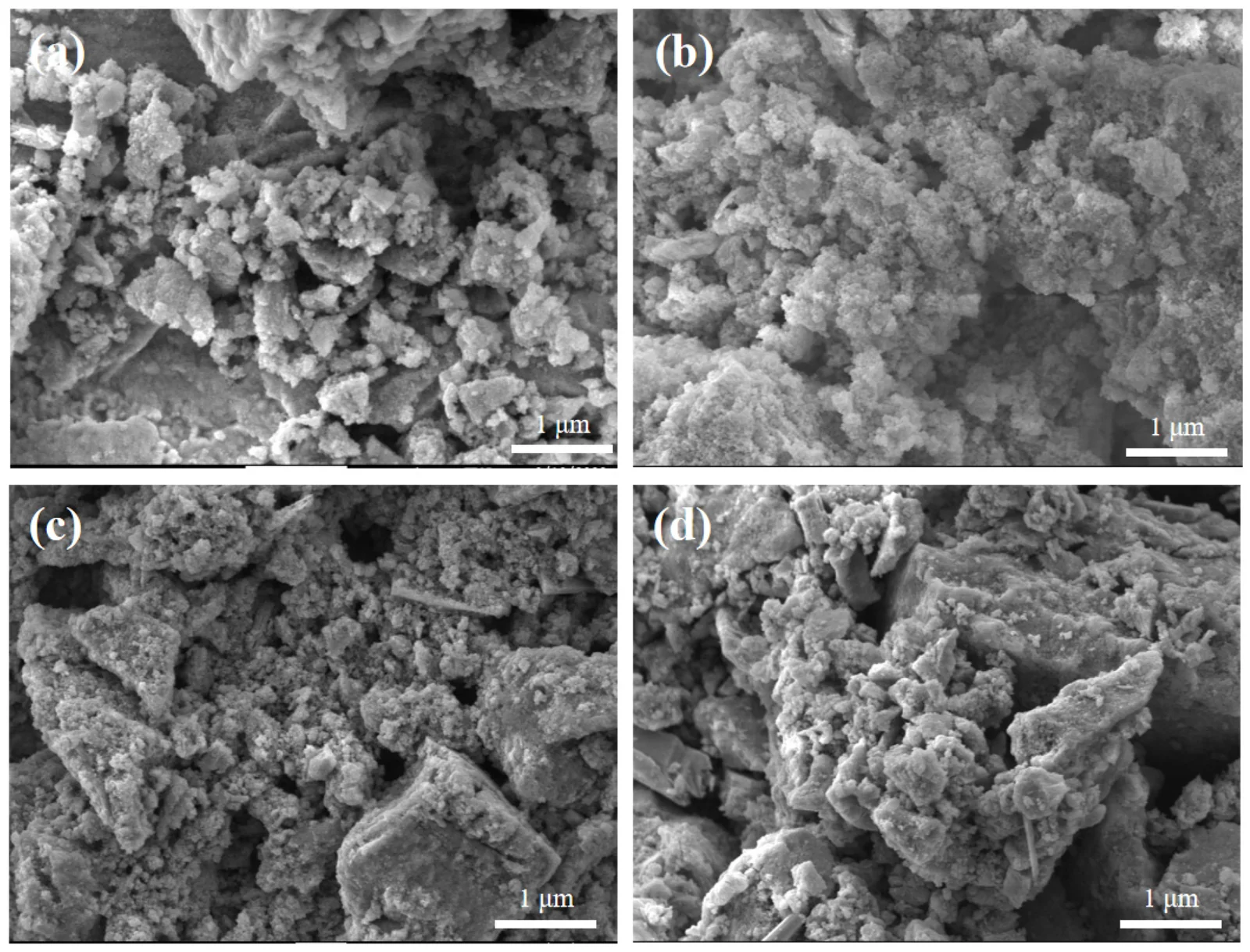
SEM images of FeMnTiO_x_ and FeMnM_0.3_TiO_x_ catalysts: (**a**) FeMnTiO_x_, (**b**) FeMnCe_0.3_TiO_x_, (**c**) FeMnCo_0.3_TiO_x_, and (**d**) FeMnW_0.3_TiO_x_.

**Figure 3:**
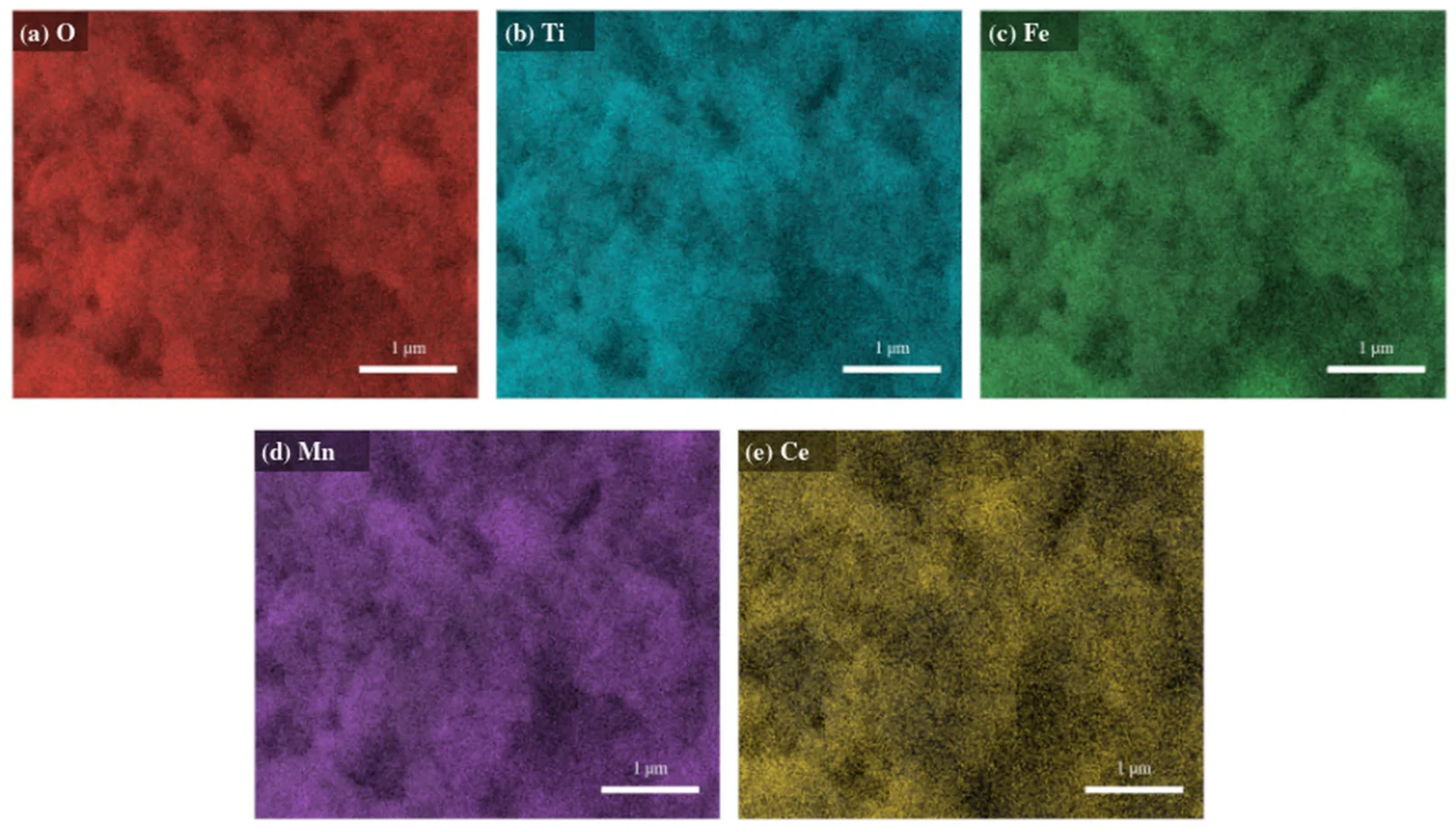
EDS elemental mapping images of FeMnCe_0.3_TiO_x_: (**a**) O, (**b**) Ti, (**c**) Fe, (**d**) Mn, and (**e**) Ce.

Figure 2 shows the morphological evolution of FeMnTiO_x_ and FeMnM_0.3_TiO_x_ (M = Ce, Co, W) catalysts observed by SEM. Figure 2a presents FeMnTiO_x_, Figure 2b FeMnCe_0.3_TiO_x_, Figure 2c FeMnCo_0.3_TiO_x_, and Figure 2d FeMnW_0.3_TiO_x_. The undoped sample displays compact aggregates with relatively large particles and limited visible interparticle voids. After Ce doping, the surface becomes rougher and contains finer particles with more apparent voids, which is consistent with the higher surface area and pore volume in Table 1. Co and W doping produce more evident bulk aggregates, consistent with their sharper TiO_2_ diffraction features and lower surface areas. Because SEM contrast alone cannot establish elemental distribution, the morphological observations are considered together with the EDS maps in Figure 3.

Figure 3 presents the EDS elemental maps of the best-performing FeMnCe_0.3_TiO_x_ catalyst. The O, Ti, Fe, Mn, and Ce signals extend throughout the analyzed particle region and show comparable spatial coverage, with no pronounced isolated Ce-rich domain in the selected field. The maps confirm the presence of the constituent elements and support their spatial co-distribution within the mapped area. This interpretation is restricted to the representative field examined by SEM–EDS.

### 2.3. N_2_ Adsorption–Desorption Analysis

**Figure 4:**
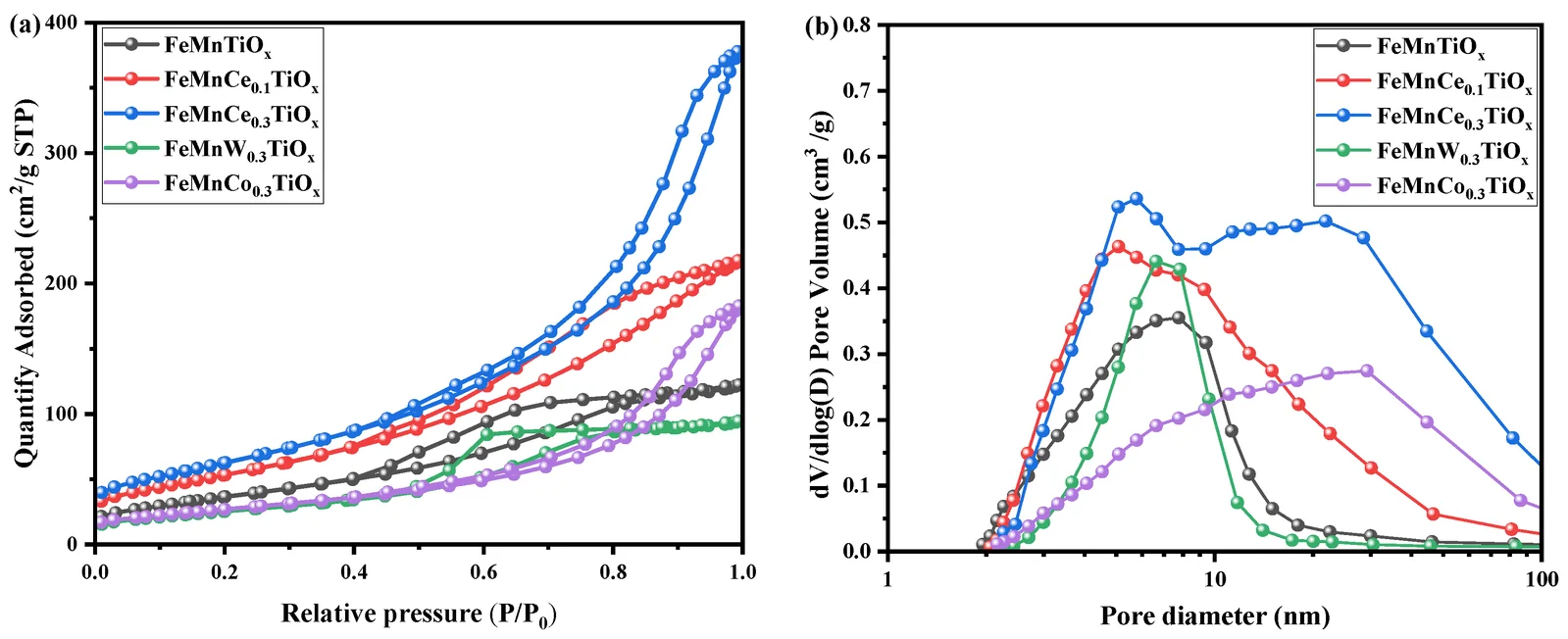
N_2_ adsorption–desorption isotherms at −196 °C (**a**) and pore size distribution curves (**b**) of FeMnTiO_x_ and FeMnMTiO_x_ catalysts (M = Ce, Co, W).

Figure 4a shows the N_2_ adsorption–desorption isotherms measured at −196 °C, and Figure 4b presents the corresponding pore-size distributions. The term “BET analysis” is used only for specific-surface-area calculation. All samples show type-IV isotherms, supporting predominantly mesoporous textures. FeMnCe_0.1_TiO_x_, FeMnCe_0.3_TiO_x_ and FeMnCo_0.3_TiO_x_ exhibit H3-like hysteresis, commonly associated with slit-like or interparticle mesopores, whereas FeMnTiO_x_ and FeMnW_0.3_TiO_x_ show H4-like loops that can also arise from narrow slit-shaped mesopores and pore-network effects [24,25]. Because no t-plot, CO_2_ adsorption or other dedicated micropore analysis was performed, the H4-like loops are not used to claim a micropore–mesopore composite. Pore-size distributions were calculated by BJH analysis of the desorption branch. These values are used comparatively because desorption-branch BJH results can be affected by pore blocking, metastability and model assumptions; NLDFT/QSDFT fitting was not performed [26]. With increasing Ce content, a pore population in the 20–30 nm range becomes more evident for FeMnCe_0.3_TiO_x_, indicating additional mesoporous channels.

Table 1 summarizes the specific surface area, total pore volume and average pore size of the catalysts. Total pore volume was determined from N_2_ uptake at P/P_0_ ≈ 0.99, and the average pore size was calculated from the desorption-branch BJH analysis. Relative to FeMnTiO_x_ (136 m^2^ g^−1^ and 0.19 cm^3^ g^−1^), Ce doping increases both surface area and pore volume, with FeMnCe_0.3_TiO_x_ reaching 231 m^2^ g^−1^ and 0.58 cm^3^ g^−1^. Co doping gives a larger pore volume than the undoped sample but a lower surface area, whereas W doping yields the smallest pore volume and a lower surface area. These are structural observations; their relationship to catalytic activity is evaluated after the catalytic data in Section 2.7.1 and Section 2.7.5.

Overall, the N_2_ adsorption–desorption results show that the dopants regulate the mesoporous texture in different ways. Ce, especially at Ce/Mn = 0.3, increases surface area, pore volume and mesopore accessibility; Co mainly changes pore volume without increasing surface area; and W maintains a more crystalline, less porous texture. These descriptors are considered together with acidity, redox behavior and the activity data below rather than being treated as stand-alone predictors.

### 2.4. XPS Analysis

**Figure 5:**
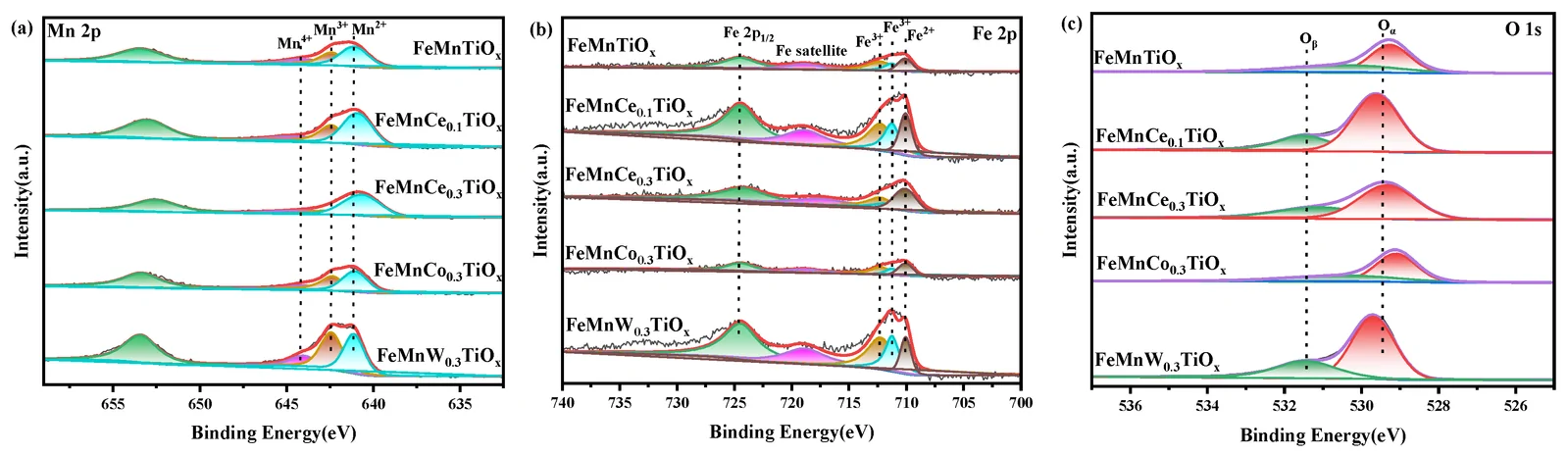
XPS spectra of the catalysts: (**a**) Mn 2p, (**b**) Fe 2p, and (**c**) O 1s.

The surface elemental composition and chemical states of the catalysts were analyzed by XPS. Figure 5a–c show the Mn 2p, Fe 2p, and O 1s spectra, respectively. The Fe 2p spectra in Figure 5b indicate the coexistence of Fe^3+^ and Fe^2+^, with Fe^3+^ as the dominant species [27]. The relative concentrations of Fe^3+^ and Fe^2+^ are listed in Table 2. Different dopants and loading levels change the Fe^3+^/Fe^2+^ balance, which can be attributed to electronic interactions among metal oxides. Because the electronegativities of Fe and the individual dopant (Ce, Co, or W) differ, electron density around Fe may be redistributed after modification, thereby affecting redox behavior and NH_3_/NO_x_ adsorption.

Dopant-specific high-resolution spectra were fitted using the same background and line-shape procedure as the Mn, Fe and O envelopes. For FeMnCe_0.1_TiO_x_, the Ce 3d envelope contained 31.8% Ce^3+^ and 68.2% Ce^4+^; for FeMnCe_0.3_TiO_x_, the corresponding fractions were 35.6% and 64.4%, indicating a slightly larger reduced-Ce fraction at the higher Ce loading. The Co 2p fit for FeMnCo_0.3_TiO_x_ gave 67.2% Co^2+^ and 32.8% Co^3+^, whereas the W 4f fit for FeMnW_0.3_TiO_x_ gave 92.1% W^6+^ and 7.9% W^5+^. These area-normalized fractions support the presence of mixed surface valence states for Ce and Co and predominantly W^6+^ for the W-modified sample; they are used as supporting evidence rather than as a direct measure of bulk stoichiometry.

An appropriate Fe^3+^ proportion is beneficial for NH_3_-SCR because it promotes redox cycling and the adsorption and activation of NH_3_/NO_x_ species. However, an excessively strong oxidation ability may also enhance nonselective NH_3_ oxidation at high temperature, leading to reduced high-temperature activity [28]. Therefore, the role of Fe^3+^ should be considered together with Mn valence distribution, surface oxygen species and acidity rather than as an isolated descriptor.

The Mn 2p spectra in Figure 5a contain Mn^2+^ (639.9–641.3 eV), Mn^3+^ (641.4–642.5 eV) and Mn^4+^ (643.0–645.0 eV) components [29,30]. Table 2 reports the area-normalized fractions of all three Mn states together with the Fe and O components. The Mn^2+^, Mn^3+^ and Mn^4+^ percentages were obtained directly from the fitted Mn 2p component areas and normalized to the total fitted Mn 2p area. The Mn^4+^/Mn^3+^ ratio increases from 0.70 for FeMnTiO_x_ to 1.25 for FeMnCe_0.3_TiO_x_, the highest value in the series. This ratio is consistent with more effective Mn^4+^/Mn^3+^ cycling and reactive-oxygen transfer, which supports the superior activity of FeMnCe_0.3_TiO_x_ despite the higher-temperature reduction features observed in H_2_-TPR [31,32].

Figure 5c shows the O 1s XPS spectra. The O 1s peak can be fitted into two components: lattice oxygen (Oα) at lower binding energy around 529.5–530.0 eV and surface adsorbed oxygen-containing species (Oβ), such as hydroxyl-related or weakly adsorbed oxygen species, at higher binding energy around 531.0–532.0 eV [33,34]. The label assignment in Figure 5c has been corrected accordingly. In view of recent discussions on O 1s interpretation, the peak near 531 eV is not directly assigned to oxygen vacancies in this work. Instead, the Oβ fraction is used as an indicator of surface oxygen-containing species that may participate in NO oxidation and interfacial oxygen transfer. A moderate Oβ proportion can promote NO oxidation to NO_2_ and facilitate the fast SCR route [35,36], whereas excessive surface oxygen species may also favor nonselective NH_3_ oxidation [37,38,39]. Therefore, the suitable Oα/Oβ balance of FeMnCe_0.3_TiO_x_ contributes to its high activity under the evaluated conditions.

### 2.5. H_2_-TPR Analysis

**Figure 6:**
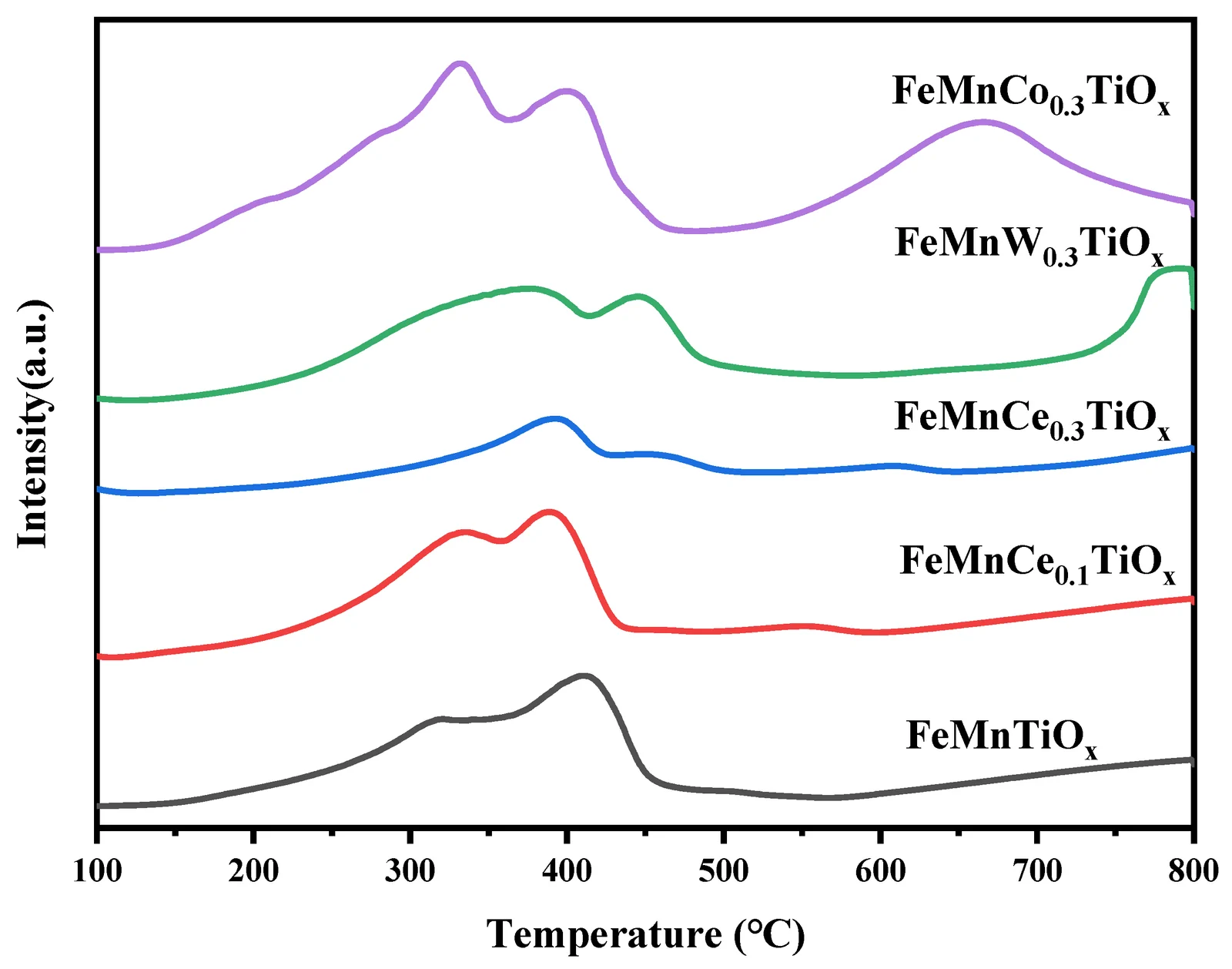
H_2_-TPR profiles of FeMnTiO_x_ and FeMnMTiO_x_ catalysts (M = Ce, Co, W).

H_2_-TPR was performed to investigate the reducibility of the catalysts. As shown in Figure 6, FeMnTiO_x_ exhibits two main reduction peaks at approximately 320 °C and 410 °C, which are attributed to the stepwise reduction of MnO_2_ to Mn_2_O_3_ and Mn_2_O_3_ to Mn_3_O_4_, respectively [40]. After Ce doping, FeMnCe_0.1_TiO_x_ shows peaks around 334 °C and 388 °C, indicating that the first reduction step shifts slightly to a higher temperature while the second step becomes easier. FeMnCe_0.3_TiO_x_ shows two peaks at approximately 391 °C and 441 °C, suggesting stronger metal–oxygen interactions and more stable dispersed oxide species at higher Ce content. This stabilization is consistent with the suppressed TiO_2_ crystallization observed by XRD and the increased surface area observed by N_2_ adsorption–desorption. Co doping shifts the reduction peaks to a lower temperature, while W doping shifts them to a higher temperature. Therefore, dopants influence reducibility in different ways: Co enhances low-temperature reducibility but does not sufficiently improve surface area or stability; W strengthens structural stability but sacrifices texture; and Ce balances structural dispersion, Mn valence regulation and oxygen transfer, leading to the best NH_3_-SCR performance. The weak residual signal above 700 °C is assigned conservatively to the reduction of bulk-like, strongly bound oxide species rather than to the surface redox sites governing low-temperature SCR [41].

### 2.6. NH_3_-TPD Analysis

**Figure 7:**
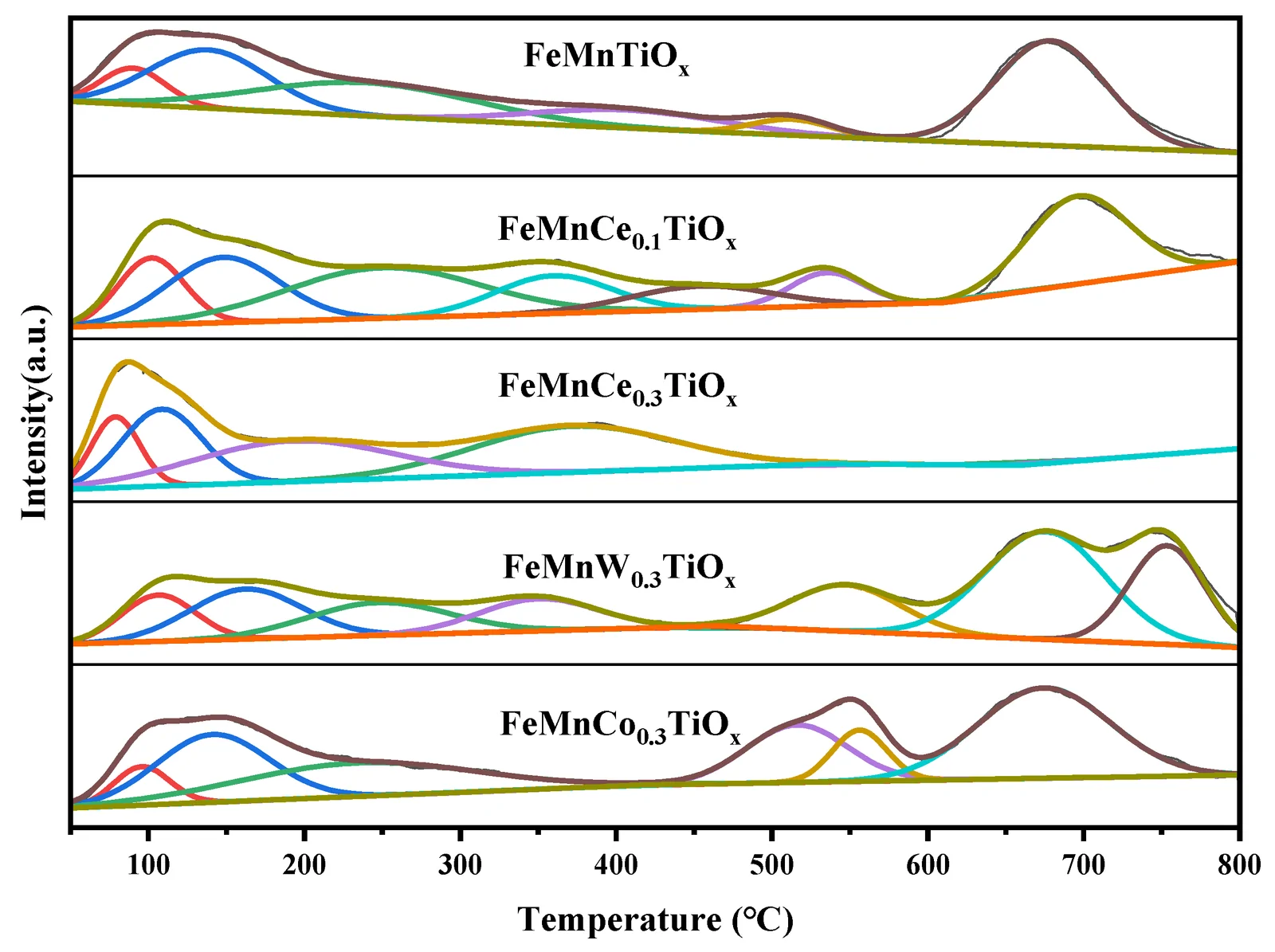
NH_3_-TPD profiles of FeMnTiO_x_ and FeMnMTiO_x_ catalysts (M = Ce, Co, W).

The surface acidity of the as-prepared catalysts was systematically characterized by NH_3_-TPD, and the fitted deconvolution curves are displayed in Figure 7. All samples show multiple NH_3_ desorption peaks, which were quantitatively divided into three categories based on peak center temperatures: weak acid sites (<200 °C), medium-strong acid sites (200–400 °C) and strong acid sites (>400 °C) [42], and the corresponding integrated peak areas for each type of acid site are summarized in Table 3. In accordance with the thermal stability difference between NH_4_^+^ bound to Brønsted acid sites and coordinatively adsorbed NH_3_ on Lewis acid sites, the low-temperature desorption peaks primarily originate from weakly adsorbed NH_4_^+^ on Brønsted acid sites, while the medium- and high-temperature desorption bands are assigned to NH_3_ anchored on Lewis acid sites [43]. Among these acid sites, medium-strong Lewis acid sites serve as the dominant active centers for low-temperature NH_3_-SCR reactions, since the adsorbed NH_3_ on such sites can be effectively activated into reactive -NH_2_ intermediates to drive the redox cycle of the SCR pathway [44,45].

Relative to the pristine FeMnTiO_x_ catalyst, heteroatom doping regulates the amount and distribution of acid sites to varying extents. Notably, Ce modification dramatically elevates the total quantity of both weak and medium-strong acid sites for FeMnCe_0.3_TiO_x_ (with peak areas of 2452.5042 and 1293.2078, respectively), and completely eliminates high-temperature strong acid sites. Such a well-balanced acid site distribution, combined with its enlarged specific surface area, abundant mesoporous channels and optimized Mn^4+^/Mn^3+^ redox ratio, synergistically endows this catalyst with superior low-temperature NO conversion efficiency. By contrast, Co and W doping also adjust the surface acidity of FeMnTiO_x_, yet FeMnCo_0.3_TiO_x_ and FeMnW_0.3_TiO_x_ possess markedly reduced medium-strong acid site amounts (321.2095 and 472.4043) alongside deteriorated porous structure and poor reducibility, which greatly restrict the enhancement of their overall catalytic performance.

The integrated areas in Table 3 are semi-quantitative relative TCD responses (a.u.), not calibrated absolute acid-site densities. No mass spectrometer or in situ infrared detector was coupled to the TPD outlet, and separate blank desorption profiles were not used to identify every evolved species. Accordingly, the weak/medium-strong/strong categories are operational temperature ranges, and their Brønsted/Lewis assignments are discussed as literature-supported tendencies rather than direct spectroscopic identification. The comparison is therefore restricted to samples measured with the same instrument program.

### 2.7. Catalytic Performance Evaluation

#### 2.7.1. Catalytic Activity

**Figure 8:**
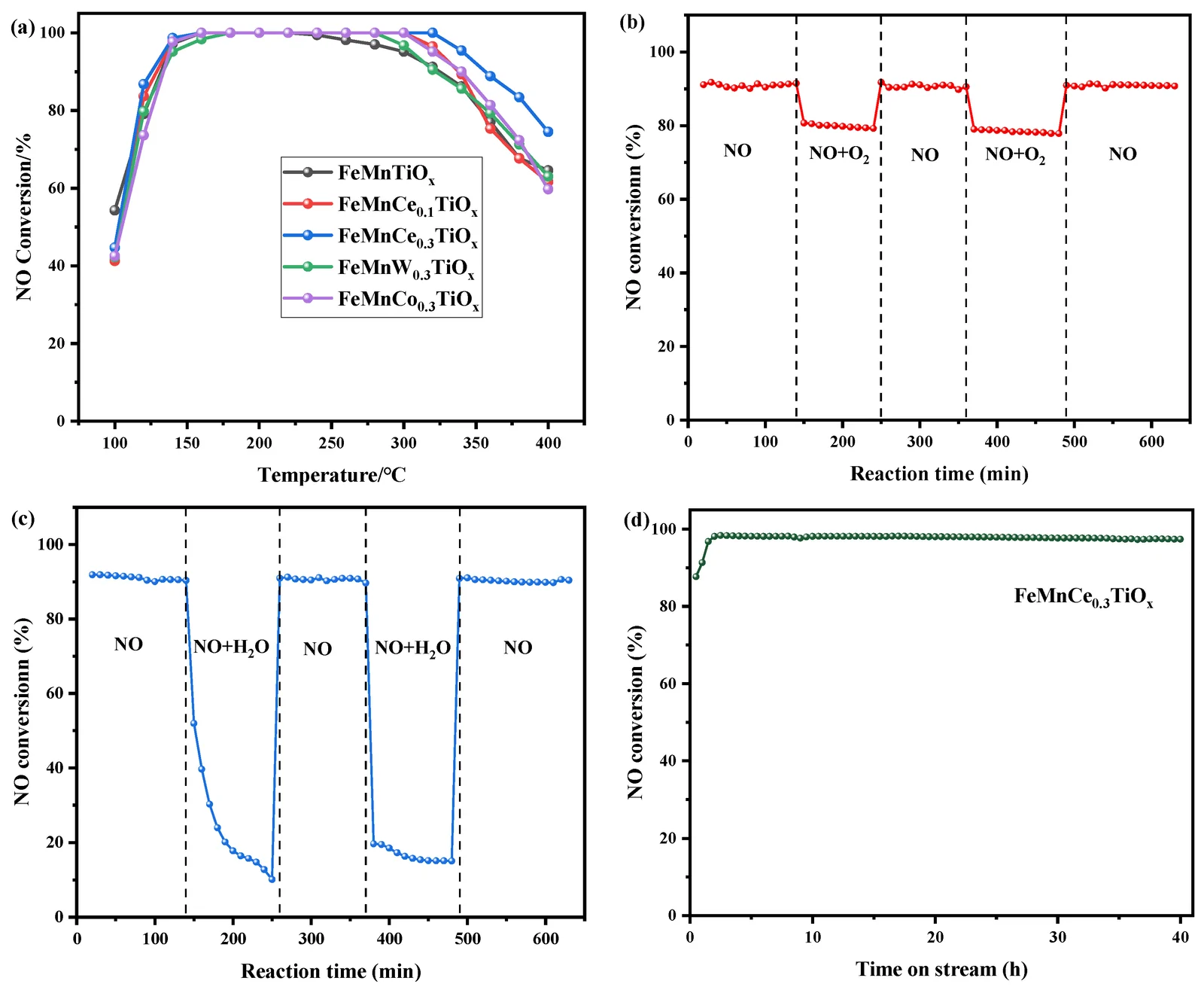
Catalytic performance of FeMnTiO_x_ and FeMnMTiO_x_ (M = Ce, Co, W): (**a**) activity; (**b**) O_2_ switching; (**c**) H_2_O switching; (**d**) dry-feed stability at 350 °C (no added O_2_/H_2_O).

Figure 8a compares the NO conversion of FeMnTiO_x_ and FeMnM_0.3_TiO_x_ catalysts. FeMnTiO_x_ reaches 98–100% conversion at 160–260 °C but decreases to 78% at 360 °C and 62% at 400 °C. FeMnCe_0.1_TiO_x_ reaches 76% at 360 °C and 60% at 400 °C. FeMnCe_0.3_TiO_x_ provides the broadest window, maintaining at least 90% conversion from 140 to 360 °C and retaining 75% at 400 °C. At 360 °C, FeMnW_0.3_TiO_x_ and FeMnCo_0.3_TiO_x_ give 78% and 82% conversion, respectively. These data show that improved reducibility alone is insufficient; the high activity of FeMnCe_0.3_TiO_x_ coincides with its higher surface area, pore volume, Mn^4+^/Mn^3+^ ratio and medium-strong acid response.

#### 2.7.2. O_2_ and H_2_O Resistance

The effects of O_2_ and H_2_O on FeMnCe_0.3_TiO_x_ were evaluated by switching experiments at 350 °C (Figure 8b,c). NO conversion was 90.8% before O_2_ addition, decreased to 78.7% with 5% O_2_, and recovered to 90.6% after O_2_ removal. Water produced a much stronger but reversible inhibition: conversion declined from 90.7% to minima of 10.3% and 15.1% during two successive 2% H_2_O exposure periods, then recovered to 90.5% and 89.9% after water removal. Thus, O_2_ causes a moderate reversible loss, whereas H_2_O strongly blocks adsorption sites under the tested condition without causing permanent deactivation over the two switching cycles.

#### 2.7.3. Catalytic Stability

The long-term stability of FeMnCe_0.3_TiO_x_ was evaluated at 350 °C under the dry base feed (500 ppm NO, 500 ppm NH_3_ and N_2_ balance), without added O_2_ or H_2_O. After the initial activation period, NO conversion averaged 97.4% and remained within 96.8–98.0% over 40 h (Figure 8d). This result establishes dry-feed time-on-stream stability and is not interpreted as H_2_O resistance; the separate switching test in Figure 8c shows strong reversible inhibition by 2% H_2_O. TG analysis of the spent catalyst showed a total mass loss of 2.8% from 30 to 700 °C, comprising 1.3% below 150 °C, 0.7% from 150 to 300 °C and 0.8% from 300 to 600 °C. TPO showed only a weak CO_2_-evolution maximum at 362 °C, corresponding to an integrated carbon amount of 0.46 wt.%. The small deposit-associated fraction is consistent with the limited deactivation observed during the 40 h dry-feed test, although longer tests under H_2_O/SO_2_-containing feeds remain necessary.

#### 2.7.4. Literature Benchmarking and Scope Limitations

Table 4 places FeMnCe_0.3_TiO_x_ alongside representative low-temperature and commercially relevant systems. Under the present protocol, FeMnCe_0.3_TiO_x_ delivers a 140–360 °C window at NO conversion ≥90%, 75% conversion at 400 °C, and a 40 h mean of 97.4% at 350 °C under the dry base feed. Fe/Co-modified Mn-Ce/TiO_2_ catalysts have reported nearly complete conversion at 150–225 °C with improved sulfur tolerance [46], while Co-doped Fe-Mn@CNTs were developed for severe H_2_O/SO_2_ feeds [27]. Supported V_2_O_5_-WO_3_/TiO_2_ remains an industrial benchmark for medium-to-high temperatures [47], and the IHC-ready Pd/Re-NiMo system reached approximately 95% NO_x_ conversion at 250 °C under induction heating [12]. Because test protocols differ, this comparison is contextual rather than a claim of commercial superiority.

A supplementary sulfur resistance test was conducted at 350 °C by introducing 100 ppm SO_2_ into the dry base feed for 8 h. NO conversion decreased from 97.2% before SO_2_ exposure to 90.4% after 8 h and recovered to 95.6% within 2 h after SO_2_ removal, indicating moderate and largely reversible sulfur inhibition under this protocol. Calibrated outlet analysis further showed that N_2_O remained below 8.5 ppm throughout the 140–360 °C high-activity window and reached 12.4 ppm at 400 °C. On the stated nitrogen-balance basis, these concentrations correspond to N_2_ selectivity of at least 98.3% from 140 to 360 °C and 97.5% at 400 °C. The sulfur test is a short screening experiment and is not presented as evidence of long-term industrial sulfur durability.

#### 2.7.5. Structure–Performance Correlation

**Figure 9:**
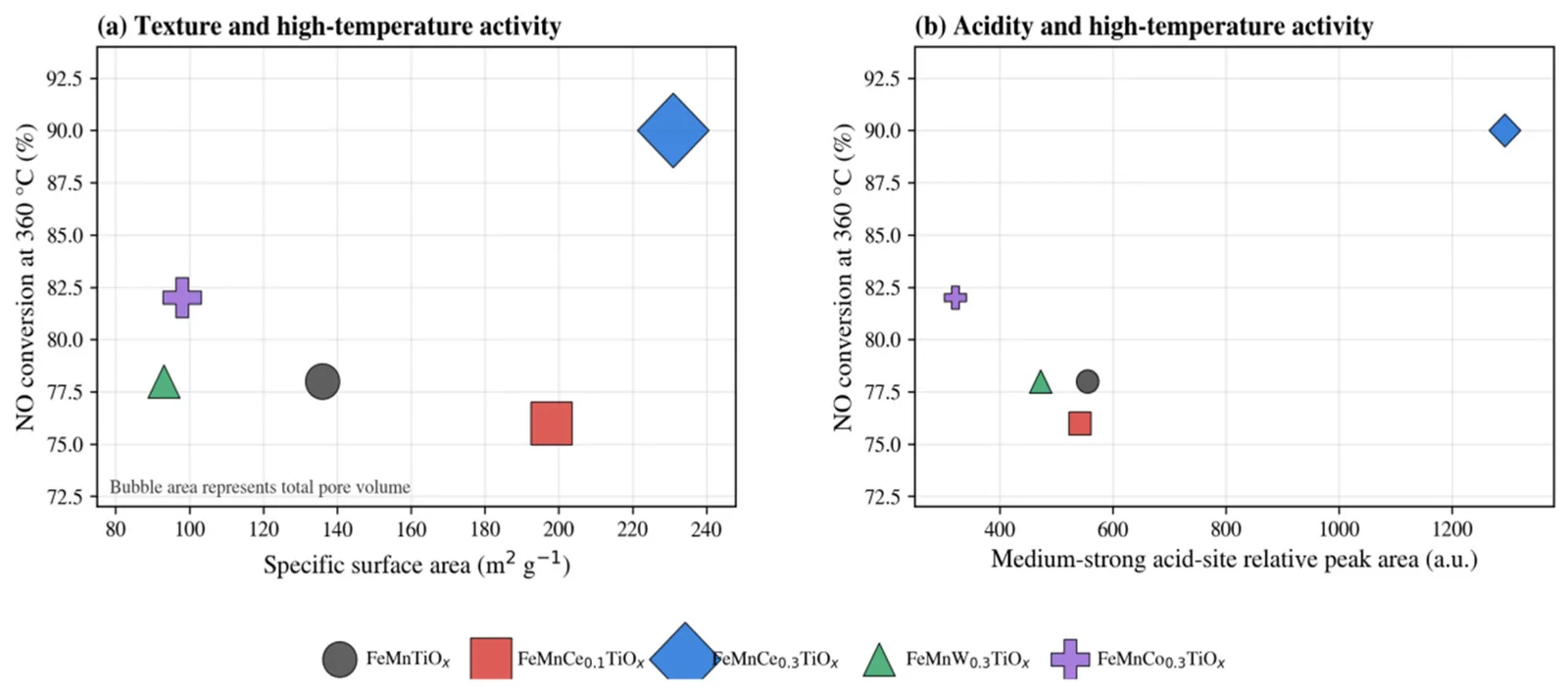
Structure–performance relationships at 360 °C: (**a**) BET area versus NO conversion (bubble area = pore volume); (**b**) medium-strong acid peak area versus NO conversion. Source: Table 1 and Table 3 and Figure 8a.

Figure 9 integrates the existing texture, acidity and activity data. FeMnCe_0.3_TiO_x_ occupies the upper-right region in both panels, combining the largest surface area and pore volume with the largest medium-strong acid relative peak area and the highest NO conversion at the plotted 360 °C point. The other samples do not follow a single monotonic descriptor-performance trend: for example, FeMnCo_0.3_TiO_x_ retains comparatively high conversion despite lower surface area and medium-strong acid area. The graphical comparison therefore supports a multi-factor interpretation in which texture, accessible acidity and redox behavior act together.

### 2.8. Mechanism Discussion

At the plotted 360 °C point, the activity order is approximately FeMnCe_0.3_TiO_x_ > FeMnCo_0.3_TiO_x_ > FeMnTiO_x_ ≈ FeMnW_0.3_TiO_x_ > FeMnCe_0.1_TiO_x_. Together with the lower-temperature plateau in Figure 8a, this order shows that the dopant effect is not governed by one property alone, but by the combined regulation of crystallinity, texture, surface redox state and acidity.

The present ex situ XRD, XPS, H_2_-TPR and NH_3_-TPD data do not directly identify surface intermediates. Accordingly, the following sequence is proposed as a literature-consistent working model rather than a uniquely proven mechanism. An Eley–Rideal route is considered dominant, with a parallel Langmuir–Hinshelwood contribution: NH_3_ adsorbs on acid sites and is activated to -NH_2_ species; gaseous NO can react with -NH_2_, while a smaller fraction of NO is oxidized/adsorbed as NO_x_ species that react with adsorbed NH_3_-derived intermediates. The overall standard-SCR stoichiometry is 4NH_3_ + 4NO + O_2_ → 4N_2_ + 6H_2_O. A plausible surface sequence is listed below.

NH_3_(g) + *acid ⇌ NH_3_* (1)

NH_3_* + O* → NH_2_* + OH* (2)

NH_2_* + NO(g) → NH_2_NO* → N_2_ + H_2_O + * (3)

NO(g) + O* ⇌ NO_x_* (4)

NH_3_*/NH_2_* + NO_x_* → N_2_ + H_2_O + * (5)

Mn^4+^ + Fe^2+^ ⇌ Mn^3+^ + Fe^3+^ (6)

The elementary pathway is not proposed to differ fundamentally among the synthesized materials. Instead, the dopants change the relative rates and availability of the steps: Ce at Ce/Mn = 0.3 most effectively combines a disordered porous texture, accessible medium-strong acidity, a favorable Mn/Fe redox balance and possible dopant-mediated electron transfer; Co promotes low-temperature reducibility but does not provide the same texture–acidity balance; and W favors a more stable, crystalline oxide framework while reducing accessible surface area. Confirmation of individual intermediates and pathway shares requires in situ/operando spectroscopy.

## 3. Materials and Methods

### 3.1. Chemicals and Reagents

The main reagents used in this study were manganese nitrate (Mn(NO_3_)_2_), iron nitrate (Fe(NO_3_)_3_·9H_2_O), cerium nitrate (Ce(NO_3_)_3_·6H_2_O), titanium sulfate (Ti(SO_4_)_2_), 75% ethanol (C_2_H_5_OH), and 25% ammonia solution (NH_3_·H_2_O), all of analytical grade. Cobalt nitrate (Co(NO_3_)_2_·6H_2_O) and ammonium metatungstate ((NH_4_)_6_H_2_W_12_O_40_·xH_2_O) were of superior grade. Reagent suppliers included Aladdin Reagent Co., Ltd. (Shanghai, China), Sinopharm Chemical Reagent Co., Ltd. (Shanghai, China), and Macklin Biochemical Technology Co., Ltd. (Shanghai, China). Deionized water was prepared by Taiyuan University of Technology.

### 3.2. Catalyst Preparation

(1) Preparation of FeMnCeTiO_x_ catalysts

**Figure 10:**
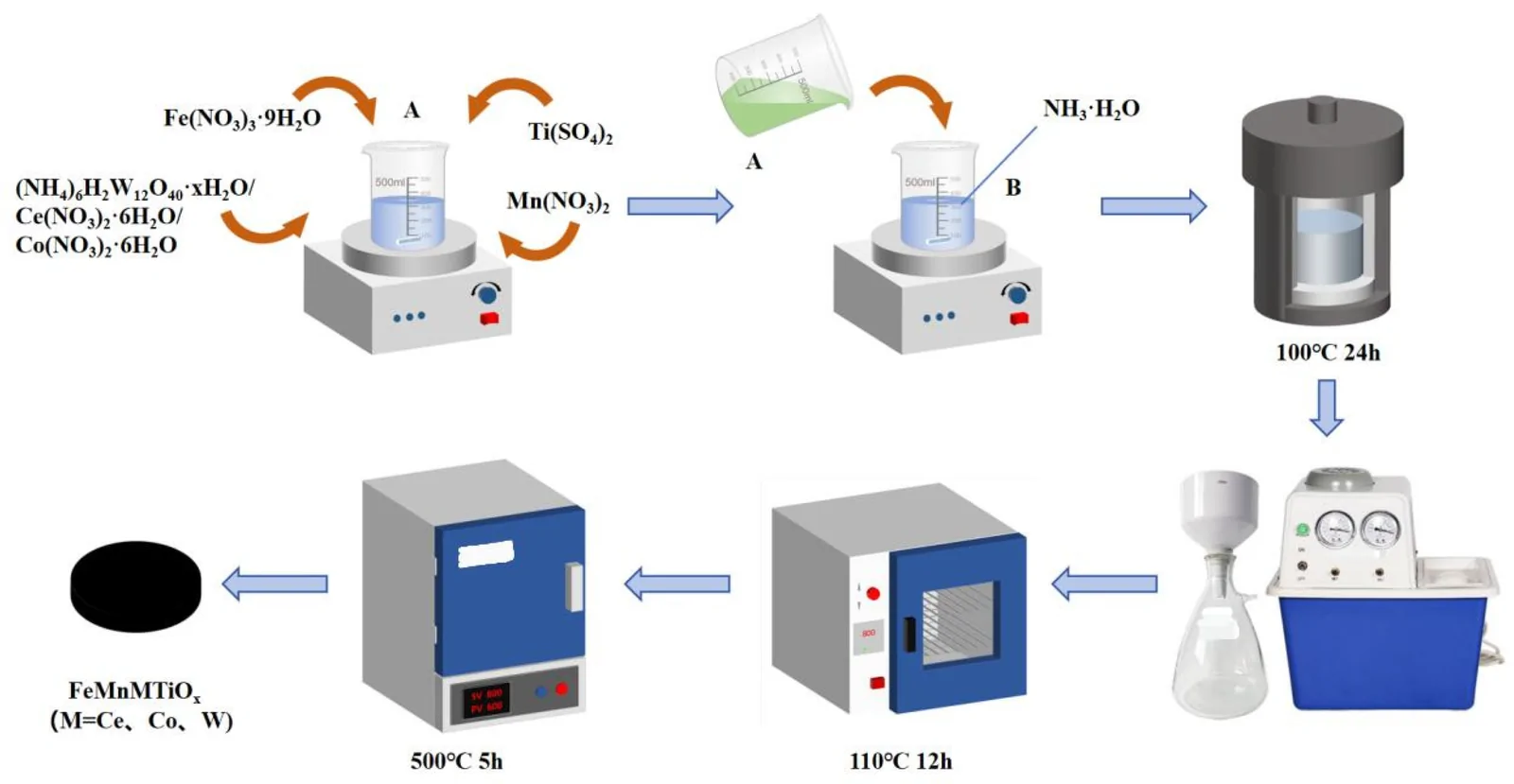
Flowchart for the synthesis of FeMnTiO_x_ and FeMnMTiO_x_ catalysts (M = Ce, Co, W).

As shown in Figure 10, firstly, 30 mL of deionized water was placed in a 50 mL beaker. Under magnetic stirring at 30 °C, 1.75 mL of 50% Mn(NO_3_)_2_ aqueous solution, 1.521 g of Fe(NO_3_)_3_·9H_2_O, a corresponding mass of Ce(NO_3_)_3_·6H_2_O (0.33 g for *n* = 0.1; 0.98 g for *n* = 0.3), and 7.2 g of Ti(SO_4_)_2_ were added sequentially according to the molar ratio Mn:Fe:Ce:Ti = 1:0.5:*n*:4 (*n* = 0.1 and 0.3). The mixture was stirred for 1 h to obtain Solution A.

Separately, 20 mL of deionized water was taken, and 26 mL of 25% concentrated ammonia solution was added, followed by stirring for 0.5 h to obtain Solution B.

Solution A was added dropwise into Solution B until the pH of the mixture reached approximately 11. The mixture was vigorously stirred at 500 rpm for 6 h, aged at 100 °C for 24 h, washed with deionized water, and filtered. The product was then dried at 110 °C for 12 h and calcined at 500 °C for 5 h, yielding FeMnCe_0.1_TiO_x_ and FeMnCe_0.3_TiO_x_, respectively.

(2) Preparation of FeMnWTiO_x_ and FeMnCoTiO_x_ catalysts

The preparation methods and elemental molar ratios of FeMnWTiO_x_ and FeMnCoTiO_x_ catalysts were identical to those of the FeMnCeTiO_x_ series, except that Ce(NO_3_)_3_·6H_2_O was replaced by the corresponding metal precursors: (NH_4_)_6_H_2_W_12_O_40_·xH_2_O (0.555 g) for FeMnW_0.3_TiO_x_ and Co(NO_3_)_2_·6H_2_O (0.655 g) for FeMnCo_0.3_TiO_x_. All other procedures were kept unchanged.

Ce was selected for a two-level loading screen because it was the principal candidate for coupled redox and textural promotion in the Fe-Mn-Ti matrix. Ce/Mn = 0.1 represents a low loading, whereas 0.3 probes a higher loading at which structural effects become evident. Co and W were fixed at M/Mn = 0.3 to compare dopant identity directly with the upper Ce loading while keeping the remaining synthesis variables unchanged. A complete loading matrix for Co and W was outside the scope of this comparative study and should be addressed in future optimization.

### 3.3. Catalyst Characterization

The crystalline phases were analyzed using an X-ray diffractometer (Rigaku Ultima IV, Akishima, Japan, Cu Kα radiation, λ = 0.15406 nm) operated at 40 kV and 40 mA. XRD patterns were recorded over 2θ = 10–80° at 5° min^−1^. Morphology was observed by SEM (Hitachi SU8010, Tokyo, Japan) at 5 kV after Au sputter coating. N_2_ adsorption–desorption isotherms were collected at −196 °C using a Micromeritics ASAP 2460 (Norcross, GA, USA) after vacuum degassing at 200 °C for 6 h. Specific surface area was calculated by BET, total pore volume from N_2_ uptake at P/P_0_ ≈ 0.99, and average pore size/pore-size distribution by BJH analysis of the desorption branch. Adsorption-branch and DFT pore-size fittings were not used. XPS spectra were collected using a Thermo Scientific ESCALAB 250Xi (Waltham, MA, USA) with Al Kα radiation (hν = 1486.6 eV), calibrated to C 1s at 284.8 eV. XPS component fitting used a Shirley background and mixed Gaussian–Lorentzian line shapes; fitted component areas within each elemental envelope were normalized to 100%. Accordingly, the Mn^2+^, Mn^3+^ and Mn^4+^ values in Table 2 and the dopant-state fractions reported in Section 2.4 are area-normalized peak-fitting results. H_2_-TPR and NH_3_-TPD were conducted on a Micromeritics AutoChem II 2920 (Norcross, GA, USA) with TCD. For both measurements, 100 mg of sample was loaded in a quartz U-tube, pretreated at 300 °C for 1 h under He (30 mL min^−1^), and cooled to 50 °C. For H_2_-TPR, 10 vol.% H_2_/Ar was supplied at 30 mL min^−1^ and the sample was heated from 50 to 800 °C at 10 °C min^−1^. For NH_3_-TPD, the pretreated sample was exposed to 10 vol.% NH_3_/He at 30 mL min^−1^ for 60 min at 50 °C, purged with He at 30 mL min^−1^ for 60 min, and then heated from 50 to 800 °C at 10 °C min^−1^ under He while desorption was monitored by TCD. All samples were tested under identical instrument programs; TPD areas are reported as relative integrated TCD responses. Approximately 10 mg of the spent catalyst was heated from 30 to 800 °C at 10 °C min^−1^ in flowing air for TG analysis. TPO used 50 mg of spent catalyst in 5 vol.% O_2_/He at 30 mL min^−1^ from 50 to 800 °C at 10 °C min^−1^, with CO_2_ monitored by a calibrated mass-spectrometric channel. SEM–EDS elemental mapping was performed on the FeMnCe_0.3_TiO_x_ sample using the EDS detector coupled to the SEM. O, Ti, Fe, Mn, and Ce maps were acquired from the same selected field and exported as individual elemental-distribution images.

### 3.4. Catalytic Activity Tests

Catalytic activity was evaluated in a fixed-bed quartz reactor using 0.20 g of catalyst (40–60 mesh). The dry base feed contained 500 ppm NO, 500 ppm NH_3_ and N_2_ as the balance; 5 vol.% O_2_ or 2 vol.% H_2_O was added only in the corresponding switching-resistance test. The total flow was 200 mL min^−1^, corresponding to approximately 60,000 mL g^−1^ h^−1^. Temperature was varied from 100 to 400 °C after steady state was reached at each point. For resistance tests, 5 vol.% O_2_ or 2 vol.% H_2_O was introduced at 350 °C and subsequently removed. The 40 h stability test was performed at 350 °C under the dry base feed without added O_2_ or H_2_O. For the supplementary sulfur screen, 100 ppm SO_2_ was introduced at 350 °C for 8 h and then removed for a 2 h recovery period. Outlet N_2_O was quantified using a calibrated FTIR channel with a reporting limit of 0.8 ppm. NO conversion was calculated as (NOin − NOout)/NOin × 100%, and N_2_ selectivity was calculated as [1 − 2N_2_Oout/(NOin + NH_3_in)] × 100%.

## 4. Conclusions and Prospects

In this study, FeMnTiO_x_ catalysts modified separately with Ce, Co, or W were prepared by coprecipitation and evaluated for low-temperature NH_3_-SCR of NO. FeMnCe_0.3_TiO_x_ achieved NO conversion ≥90% from 140 to 360 °C, retained 75% at 400 °C, and averaged 97.4% during a 40 h dry-feed test at 350 °C. Relative to FeMnTiO_x_, Ce/Mn = 0.3 increased surface area from 136 to 231 m^2^ g^−1^, pore volume from 0.19 to 0.58 cm^3^ g^−1^, Mn^4+^/Mn^3+^ from 0.70 to 1.25, and the medium-strong acid relative peak area from 555.4 to 1293.2 a.u. W produced a more crystalline, less porous structure, whereas Co improved reducibility without the same texture–acidity balance. The ex situ evidence supports a working model dominated by an Eley–Rideal route with an auxiliary Langmuir–Hinshelwood contribution. The separate H_2_O switching test showed strong reversible inhibition, so the 40 h dry-feed result is not presented as water-resistance evidence. The short 100 ppm SO_2_ screen retained 90.4% conversion after 8 h and recovered to 95.6% after removal; N_2_ selectivity remained ≥98.3% within the 140–360 °C window; and post-reaction TG/TPO indicated 0.46 wt.% carbon. These screening results do not replace longer durability and product-selectivity tests under realistic multi-component feeds.

## Figures and Tables

**Table 1:** Specific surface area calculated by the BET model and pore structure results for FeMnTiO_x_ and FeMnMTiO_x_ catalysts (M = Ce, Co, W).

Catalyst Sample	Specific Surface Area (m^2^/g)	Pore Volume (cm^3^/g)	Average Pore Size (nm)
FeMnTiO_x_	136	0.19	4.97
FeMnCe_0.1_TiO_x_	198	0.34	6.60
FeMnCe_0.3_TiO_x_	231	0.58	9.53
FeMnW_0.3_TiO_x_	93	0.15	6.09
FeMnCo_0.3_TiO_x_	98	0.28	11.51

**Table 2:** Surface elemental valence-state distribution obtained from area-normalized XPS peak-fitting results for FeMnMTiO_x_ catalysts.

Catalyst Sample	Mn^2+^ (%)	Mn^3+^ (%)	Mn^4+^ (%)	Fe^2+^ (%)	Fe^3+^ (%)	Oα (%)	Oβ (%)
FeMnTiO_x_	49.37	29.75	20.88	29.27	70.73	70.73	29.27
FeMnCe_0.1_TiO_x_	56.17	30.13	13.70	27.24	72.76	25.41	74.59
FeMnCe_0.3_TiO_x_	68.92	13.79	17.29	44.30	55.70	33.16	66.84
FeMnCo_0.3_TiO_x_	45.64	37.72	16.64	34.61	65.39	36.00	64.00
FeMnW_0.3_TiO_x_	39.95	42.67	17.38	19.03	80.97	29.05	70.95

**Table 3:** Peak areas of different strength acid sites from NH_3_-TPD deconvolution for modified FeMnTiO_x_ catalysts.

Catalyst Sample	Total Area of Weak Acid Sites	Total Area of Medium-Strong Acid Sites	Total Area of Strong Acid Sites
FeMnTiO_x_	703.9587	555.3536	1234.8052
FeMnCe_0.1_TiO_x_	444.3446	541.7023	558.1906
FeMnCe_0.3_TiO_x_	2452.5042	1293.2078	0.0000
FeMnW_0.3_TiO_x_	500.9400	472.4043	1414.1129
FeMnCo_0.3_TiO_x_	394.9174	321.2095	834.9145

**Table 4:** Contextual comparison of FeMnCe_0.3_TiO_x_ with representative NH_3_-SCR and induction-heated deNO_x_ systems.

Ref.	Scope and Distinguishing Feature	Representative Reported Performance	Catalyst/System
This work	Same-matrix dopant comparison; separate O_2_/H_2_O switching; 100 ppm SO_2_ screen; N_2_O-resolved selectivity; 40 h dry-feed stability	NO conversion ≥ 90% at 140–360 °C; 90.4% after 8 h SO_2_; N_2_ selectivity ≥ 98.3% at 140–360 °C; 97.4% mean over 40 h	FeMnCe_0.3_TiO_x_
[46]	Low-temperature activity with reported SO_2_-tolerance improvement	Nearly 100% NO conversion at 150–225 °C	Fe/Co-doped Mn-Ce/TiO_2_
[27]	Designed for high H_2_O/SO_2_ resistance; conditions differ markedly	Activity retained during a 192 h severe H_2_O/SO_2_ test	Co-doped Fe-Mn@CNTs
[47]	Commercial maturity; comparatively limited low-temperature activation	Established medium-/high-temperature NH_3_-SCR benchmark	V_2_O_5_-WO_3_/TiO_2_
[12]	Direct induction heating; noble-metal/metallic structured catalyst	Approximately 95% NO_x_ conversion at 250 °C	Pd/Re-NiMo (IHC)

## Data Availability

The original contributions presented in this study are included in the article. Further inquiries can be directed to the corresponding author.
